# *In vivo *measurement of tumor estradiol and Vascular Endothelial Growth Factor in breast cancer patients

**DOI:** 10.1186/1471-2407-8-73

**Published:** 2008-03-18

**Authors:** Stina Garvin, Charlotta Dabrosin

**Affiliations:** 1 Linköping University, Division of Oncology, Faculty of Health Sciences, University Hospital, SE-581 85 Linköping, Sweden

## Abstract

**Background:**

Angiogenesis, crucial for tumor progression, is a process regulated in the tissue micro-environment. Vascular endothelial growth factor (VEGF) is a potent stimulatory factor of angiogenesis and a negative prognostic indicator of breast cancer. VEGF is biologically active in the extracellular space and hitherto, there has been a lack of techniques enabling sampling of angiogenic molecules such as VEGF *in situ*. The majority of breast cancers are estrogen-dependent, and estrogen has been shown to regulate VEGF in normal breast tissue and experimental breast cancer. We investigated if microdialysis may be applicable in human breast cancer for sampling of extracellular VEGF *in situ *and to explore if there is an association with local estradiol and VEGF levels in normal and cancerous breast tissue.

**Methods:**

Microdialysis was used to sample VEGF and estradiol in tumors and adjacent normal breast tissue in postmenopausal breast cancer patients. VEGF and estradiol were also measured in plasma, and immunohistochemical staining for VEGF was performed on tumor sections.

**Results:**

We show that *in vivo *levels of extracellular VEGF were significantly higher in breast cancer tumors than in normal adjacent breast tissue. There was a significant positive correlation between estradiol and extracellular VEGF in normal breast tissue. However, no correlation was detected between estradiol and VEGF in tumors or between tumor VEGF and plasma VEGF.

**Conclusion:**

We conclude that VEGF and estradiol correlates significantly in normal breast tissue. Microdialysis may be used to provide novel insight in breast tumor biology and the regulation of molecules in the extracellular space of human breast tumors *in vivo*.

## Background

Vascular endothelial growth factor (VEGF) is a potent mediator of tumor angiogenesis, including neovascularization in human breast cancer [1]. In breast cancer patients, high tumor VEGF levels, as assessed by immunohistochemistry or quantitative immunoassay, appear to correlate with poor prognosis and decreased overall survival for both node-positive and node-negative breast cancer patients [2-4]. VEGF exists in several isoforms; the shorter isoforms are diffusible proteins whereas the longer isoforms are sequestered in the extracellular matrix [5]. Proteolytic cleavage may convert the longer VEGF isoforms into soluble, bioactive forms in the extracellular space where they become available to endothelial cells [5]. Sex steroids have been shown to increase VEGF expression in normal breast tissue as well as in experimental breast cancer, both *in vitro *and *in vivo *[6-10], and an estrogen responsive element (ERE) has been identified in the promoter region of the gene for VEGF [11].

Estrogen exposure is considered a major risk factor for development of breast cancer and the majority of breast cancers maintain their hormonal dependency [12,13]. After menopause the circulating levels of estradiol are greatly reduced, yet postmenopausal women have been shown to maintain breast tissue estradiol levels higher than corresponding plasma levels [14]. Moreover, levels of estradiol in breast tumor tissue have been shown to be higher than those in normal breast tissue distant from the tumor [14,15], and the major enzymes involved in estrogen synthesis – aromatase, sulphatase, and 17β-hydroxysteroid dehydrogenase – have been demonstrated in human breast cancer [16-18]. As VEGF may be released and estradiol metabolized locally in the breast, an *in situ *technique for surveying these molecules may provide novel insight into their regulation in tumor tissue *in vivo*. We have previously used microdialysis for *in vivo *measurements of estradiol, VEGF, and other molecules in normal human breast and experimental breast cancer [6-8,19-21]. In this study we have performed microdialysis in breast cancer patients in order to sample estradiol and VEGF locally in breast cancer tumors and in adjacent normal breast tissue. We found that extracellular levels of VEGF were significantly higher in tumors than in normal breast tissue *in vivo*. In addition, we show a positive correlation between normal breast tissue and plasma levels of estradiol and extracellular VEGF in normal breast tissue *in vivo*.

## Methods

### Subjects

Ten postmenopausal women, ages 51–86, participated in the study. Postmenopausal status was defined by having no spontaneous menstrual bleeding for at least a year in women older than 50. None of the women had ongoing hormonal treatment. The study was approved by the local ethical review board in Linköping, Sweden, (reference number 03–081) and performed in compliance with the Helsinki Declaration, and all women gave informed consent. Patient characteristics are provided in Table 1.

**Table 1:** Patient and tumor characteristics

	1	2	3	4	5	6	7	8	9	10
Age	67	59	51	86	83	66	56	68	65	57
Histology	lobular	ductal	ductal	ductal	ductal	ductal	ductal	lobular	lobular	ductal
Size (mm)	18	9	20	60	20	20	17	15	50	21
Disease stage (T)	1	1	1	3	2	2	1	1	2	2
Histological grade (NHG)	2	2	3	3	2	2	2	2	2	3
ER (%)	50	80	100	80	100	80	100	75	75	75
PR (%)	100	10	0	80	100	0	0	0	50	0

*Abbreviations: *ER = estrogen receptor, PR = progesterone receptor

### Microdialysis

Microdialysis was performed on the day before or the same day as surgery using microdialysis catheters with a molecular weight cut-off of 100,000 Da (CMA Microdialysis AB, Stockholm, Sweden). Each catheter consists of a tubular dialysis membrane (length 10 mm; diameter 0.52 mm) attached to a double-lumen tube (length 100 mm; diameter 0.8 mm). Prior to microdialysis, mepivacaine (0.3 mL; 5 mg/mL) was administered intracutaneously as a local anesthetic. Two microdialysis catheters were thereafter inserted in the affected breast, one intratumorally and one in adjacent, macroscopically normal breast tissue. The insertion was guided by catheters for intravenous use (Venflon, 1.4 mm; BOC Ohmeda AB, Helsingborg, Sweden). The microdialysis catheters were connected to a microinfusion pump (CMA 107, CMA Microdialysis AB) and perfused with NaCl (154 mM) and dextran-70 (40 g/L) at a rate of 0.5 μL/min. After an equilibration period of 30 minutes, the outgoing dialysate was collected and stored together with plasma samples at -70°C for subsequent analysis. No complications occurred during or after the microdialysis experiments.

Microdialysis allows sampling of the extracellular fluid by passive diffusion of substances over a semi-permeable membrane. The recovery i.e. the amount of substances that diffuse into the perfusion fluid depends on the tissue and membrane properties, the flow rate, and the size of the compound of interest [22]. Diffusion of low molecular substances over the dialysis membrane is almost complete at low flow rates using a 30 mm long dialysis membrane [23]. However, for larger molecules the recovery over the membrane decreases and the measured levels in the microdialysis sample may not be absolute concentrations in the tissue. The recovery of certain substance may be measured *in vitro*, however, this *in vitro *recovery can only be an estimate of the *in vivo *recovery since other factors such as tissue pressure and temperature will affect the diffusion of substances. For the *in vivo *recovery several calibration techniques such as the no net flux technique, the stop flow techniques, and the endogenous reference technique have been developed for low molecular substances unbound in the extracellular space. However, when measuring high molecular weight proteins these techniques may not be applicable because the proteins of interest may be bound to binding proteins *in vivo*, recombinant proteins may not have the same properties as the natural proteins *in vivo*, and plasma and tissue levels of the protein are not equal. We have previously found that in the case of VEGF tissue homogenate, unlike plasma VEGF, correlate significantly with VEGF sampled by microdialysis. We have previously reported an *in vitro *recovery of VEGF of 8% and an *in vitro *recovery of estradiol from plasma of 30% and these data were confirmed in the present study [7,20]. In the present study all microdialysis values are given as original raw data without any re-calculations.

### Quantification of estradiol and VEGF

Measurements of estradiol in plasma and microdialysis dialysate were conducted using a commercial quantitative immunoassay kit (estradiol ELISA, DRG Instruments GmbH, Marburg, Germany) according to the manufacturer's instructions. VEGF was quantified using a commercial quantitative immunoassay kit for human VEGF (Fluorokine MAP human VEGF kit, R&D systems, Abingdon, UK) and analyzed by the Luminex 100 IS System (Luminex B.V., Oosterhout, Netherlands). According to the manufacturer, the VEGF kit measures the VEGF_165 _and VEGF_121 _isoforms, and the mean minimum detectable dose is 0.81 pg/mL. The intra-assay precision for this kit is 3.7–7% which was confirmed during the experiment. All microdialysis values are stated as raw data.

### Immunohistochemistry of tumor sections

Formalin-fixed, paraffin-embedded tumors were sectioned, deparaffinized and subjected to anti-human VEGF immunohistochemistry (monoclonal mouse anti-human VEGF, specific for the VEGF_165 _and VEGF_121 _isoforms, dilution 1:20, R&D systems, with Envision detection, DAKO). Sections were counterstained with Mayer's hematoxylin. Negative controls did not show staining. In a blinded manner, entire sections were first scanned to determine the range of intensity of the staining. VEGF staining was thereafter scored as weakly or strongly positive. Estrogen receptor (ER) and progesterone receptor (PR) were determined by immunohistochemical staining using the Ventana Benchmark system (Ventana Medical Systems Inc., Arizona, USA) and defined as the fraction of positive nuclei. Monoclonal rabbit anti-human estrogen receptor alpha antibody (prediluted 1:50, Lab Vision Ltd, Suffolk, UK) and monoclonal mouse anti-human progesterone receptor antibody (prediluted 1:50, Novocastra Laboratories Ltd, Newcastle, UK) were used for staining.

### Clinicopathological data

Tumor histology, size, stage (T of the TNM classification), and Nottingham histological grade (NHG) according to Elston Ellis scoring system were determined at the Department of Pathology and Cytology, University Hospital of Linköping.

### Statistical analysis

Shapiro-Wilks' W test for normality was used. Wilcoxons' signed rank test for paired observations was used and normally distributed data was calculated using Pearson's correlation coefficient. Results are given in median [25–75 precentile].

## Results

### Characteristics of patients and tumors

Ten patients were included in the study, 7 with invasive ductal carcinoma and 3 with invasive lobular carcinoma. All of the tumors were ER-positive; 5 of 10 were PR-positive. Tumors were stage pT1-3 (TNM) and NHG 2–3. All tumors showed positive scoring for mitosis (not shown). Clinicopathological data is provided in Table 1.

### Extracellular VEGF significantly higher in breast cancer tumors than in adjacent normal breast tissue

**Figure 1:**
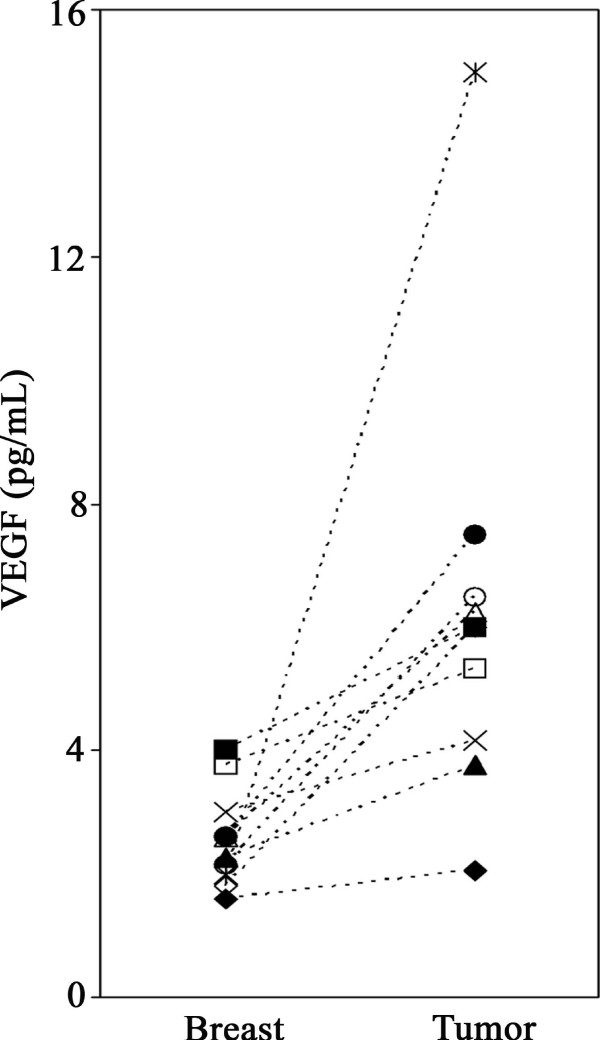
**Extracellular VEGF in normal breast and tumors of postmenopausal breast cancer patients**. VEGF sampled *in vivo *by microdialysis was significantly higher in tumors than in adjacent normal breast tissue (*p *= 0.005). Each symbol represents an individual patient.

**Figure 2:**
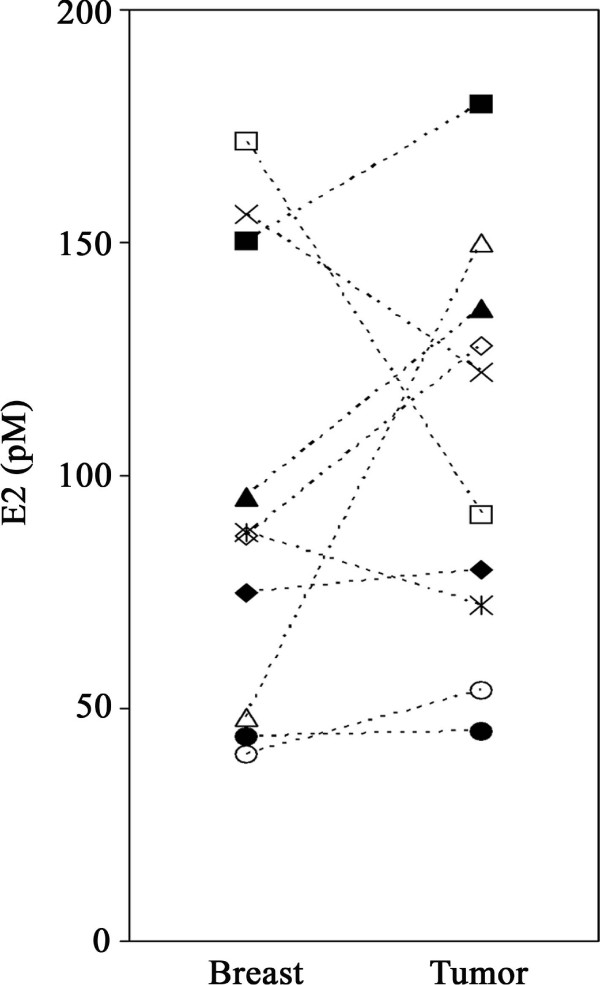
**Extracellular estradiol in normal breast and tumors of postmenopausal breast cancer patients**. No significant differences were found between the levels of estradiol in tumor and normal breast tissue sampled *in vivo *by microdialysis. Each symbol represents an individual patient.

Using microdialysis, levels of estradiol and extracellular VEGF were measured in tumors and in adjacent normal breast tissue *in vivo*. Levels of intratumoral VEGF were significantly higher than in normal breast tissue, 6.0 pg/mL [4.1–6.5] in tumors compared to 2.4 pg/mL [2,3] in breast tissue, *p *= 0.005, Wilcoxon signed rank test, Figure 1). Plasma levels of VEGF were 3.67 pg/mL [0.8–5.7], range 0.62–7.59 pg/mL. In 7 of the 10 patients tumor estradiol levels were higher than in normal breast tissue, however, when comparing the whole group no significant differences were found between the levels of tumor estradiol, 106 pM [72–136], and normal breast tissue estradiol, 87 pM [48–150], Figure 2. Plasma levels of estradiol were 60 pM [40–84], range 24–198 pM.

### Significant correlation between normal breast estradiol and breast VEGF in vivo

**Figure 3:**
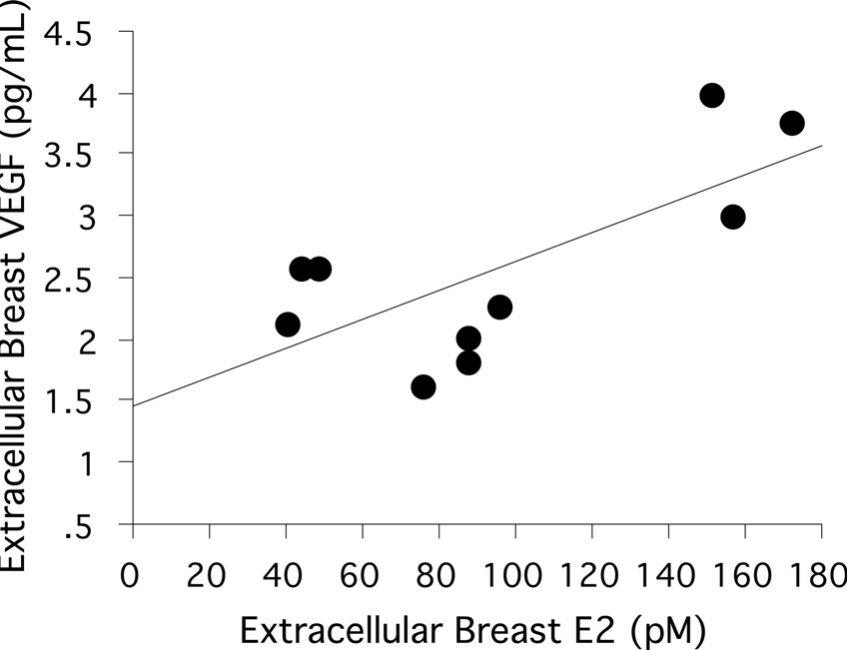
**Correlation of local extracellular breast VEGF and estradiol in normal breast tissue of breast cancer patients**. There was a significant correlation between extracellular local breast estradiol (E2) and VEGF in normal breast tissue of breast cancer patients, (r = 0.708, *p *= 0.022).

A significant positive correlation was found between plasma levels and normal breast tissue levels of estradiol, Figure 3. No correlations were found between tumor estradiol and tumor VEGF or between plasma VEGF and tumor VEGF. ER, PR, and tumor size did not correlate with VEGF or estradiol measured in microdialysis dialysate or plasma.

### Immunohistochemistry of VEGF

All tumor sections showed positive intracellular staining for VEGF. Inter-individual differences in intratumoral VEGF quantified in microdialysis dialysate were not reflected in the intensity of staining for VEGF.

## Discussion

To our knowledge this is the first report of intratumoral sampling of extracellular estradiol and VEGF *in vivo *in breast cancer patients. We show that tumor levels of extracellular VEGF were significantly higher than those in adjacent normal breast tissue *in vivo*. In addition, we show a positive correlation between normal breast tissue levels of estradiol and extracellular VEGF in normal breast tissue *in vivo*, in agreement with previous findings in a larger cohort of healthy women [6].

Higher extracellular VEGF in tumor tissue compared to normal tissue is in line with previous *ex vivo *findings demonstrating an up-regulation of VEGF in breast cancer compared to adjacent non-neoplastic tissue [24,25]. The inter-individual differences in extracellular VEGF measured *in vivo *were not apparent in the *ex vivo *immunohistochemical staining. This may be explained by the fact that the shorter soluble isoforms of VEGF, which are sampled using microdialysis, are not likely to be detected in the extracellular space by immunostaining of tissue sections [26]. The freely diffusible VEGF_121 _isoform has been shown to be more angiogenic and tumorigenic than other splice variants of VEGF and is considered the predominant isoform in human breast cancer [26,27]. Moreover, our previous studies suggest that quantitative differences in secreted VEGF are not necessarily detectable in semi-quantitative immunohistochemical staining [6,8,9]. Hence, sampling VEGF in the extracellular space, where it exerts its effects, may add significant insight into its regulation in tumor tissue.

Previous *ex vivo *studies have shown that estradiol levels may be higher in tumor tissue than in normal breast tissue distant from the tumor [14,15]. The demonstration of enzymes involved in estrogen synthesis in human breast cancer has led to the intracrine concept that local estrogens produced in a breast tumor and surrounding tissue contributes to tumor development and progression [16-18]. In this study, estradiol levels measured in tumors *in vivo *were indeed higher than in normal breast tissue in 7 of the 10 patients with up to three times higher levels of estradiol in tumors, supporting possible intratumoral estrogen production in some breast cancers.

While a significant correlation was found between normal tissue estradiol and VEGF, there was no correlation between levels of tumor estradiol and VEGF. Although all tumors were ER positive, levels of expression varied between patients and half of the tumors showed no PR expression. Loss of PR expression has previously been attributed to a non-functional ER [28], which may be one reason for the absence of correlation between tumor estradiol and tumor VEGF. Therefore our results do not rule out a possible regulation of VEGF by estradiol in breast cancer tumors. Hypoxia is a potent regulator of VEGF and tumor angiogenesis via hypoxia inducible factor-I [29,30], and we have previously demonstrated a positive correlation between tumor size and VEGF in experimental breast cancer models [8,31]. Xenografts derived from cell lines are however more homogeneous than human tumors due to the use of only one clone of cancer cell [32]. In this study both lobular and ductal carcinomas were included, and tumor size varied between 9–60 mm. The heterogeneity and the small sample size may explain, at least in part, the lack of correlation between tumor size and tumor VEGF.

Plasma and tumor levels of VEGF did not correlate in this study in line with our previous findings demonstrating that less than half of the VEGF in circulating plasma originates from the tumor [7]. This suggests that determination of VEGF in plasma is a poor indicator of tumor VEGF.

The fact that estradiol may be synthesized and VEGF mobilized locally in the breast and in breast cancer emphasizes the need to sample these molecules in the extracellular space. This study is the first of its kind to measure VEGF and estradiol in human breast cancer tumors *in situ *by microdialysis. Sampling regulators of the tumor microenvironment directly *in situ *is highly desirable taking into consideration that gene expression levels and intracellular protein levels are not always indicative of biologically active extracellular protein levels [8]. It may be argued that the differences seen between tumor and normal tissue merely reflect differences in recovery between the tissues. However, tumor tissue have increased blood flow compared to normal breast tissue which in theory would decrease the recovery and result in decreased levels of the compounds in the tumor. In our present study tumor tissue exhibited higher levels of VEGF. Hence, these results cannot be explained by possible differences of the *in vivo *recovery only. Moreover, correlations are measured in the same sample from the same tissue and these results are unaffected by different tissue recoveries. While studies today are generally limited to *in vitro *cell culture methods, animal models, and *ex vivo *surgical specimens, microdialysis is emerging as unique tool for *in vivo *sampling in accessible tumors such as those of the breast [33].

## Conclusion

Our findings show that the *in vivo *levels of extracellular VEGF were significantly higher in breast cancer tumors than in normal breast tissue. In addition, our results support the role of estradiol as a potent regulator of VEGF in normal breast tissue *in vivo*. Further studies are warranted to investigate the role of VEGF in mediating sex steroid effects on breast cancer development and progression. Novel *in situ *data provided by microdialysis may increase our understanding of breast cancer tumor biology.

## Competing interests

The author(s) declare that they have no competing interests.

## Authors' contributions

CD conceived and designed the study. Microdialysis and data collection were performed by CD and SG. SG performed data analysis and SG and CD co-wrote the manuscript. Both authors read and approved the final manuscript.

## Pre-publication history

The pre-publication history for this paper can be accessed here:


